# Biofouling and Antifouling: Interactions between Microbes and Larvae of Invertebrates

**DOI:** 10.3390/ijms24076531

**Published:** 2023-03-31

**Authors:** Sergey Dobretsov, Daniel Rittschof

**Affiliations:** 1 Department of Marine Science and Fisheries, College of Agricultural and Marine Sciences, Sultan Qaboos University, Al-Khoud, P.O. Box 34, Muscat 123, Oman; 2 Nicholas School of the Environment, Duke University, 135 Duke Marine Lab Road, Beaufort, NC 28516, USA; ritt@duke.edu

## 1. Introduction: Biofouling and Antifouling

The biofouling process refers to the undesirable accumulation of micro- and macro-organisms on manufactured surfaces. Any clean substratum submerged in a marine environment is quickly fouled, first from the surface film with proteins, lipids, and other organic molecules. The process continues as surface-active molecules partition onto the surface [1,2]. Depending on conditions, and usually very soon after, microbes (referred to as microfouling) and propagules of macro-organisms (macrofouling) populate the substratum [3]. The process of biofouling lasts hours, days, months, and even years, and depends on the location, environmental conditions, and mitigation procedures. Microorganisms attach to the substratum and form biofilms [4]. Biofilms in the marine environment are composed of different species of bacteria, archaea, fungi, and prokaryotes [5]. Macrofouling organisms can be pioneers attaching as quickly as bacteria or successional, depending upon the establishment of microbes or biofilms [6].

Biofilms can induce the settlement of propagules of macro-organisms [5], inhibit their settlement [7], or have no activity. The effect of the biofilms depends on the microbial species composition, their proportion, species of larvae of invertebrates, and chemical compounds produced by biofilms.

Biofouling developed on marine installations leads to increased costs due to biodeterioration and the stress of structural materials and equipment, loss of installation buoyancy, corrosion, and high fuel consumption [8]. Thus, many marine installations are protected from biofouling using antifouling management coatings. Traditionally, antifouling is based on the use of toxic biocides, such as copper [8]. These compounds kill marine organisms, accumulate in sediments, and transfer through the food chain. Thus, non-toxic solutions are needed. These might include biomimetic surfaces, which mimic the naturally existing ones of mussels, sharks, and sea stars [9]. Another approach is to use low adhesion, non-stick surfaces that prevent the attachment or reduce the adhesion of fouling organisms [10].

However, alternative antifouling methods are not as effective or work for shorter intervals than biocidal coatings and require cleaning. Recent laboratory studies of common components of foul-release polymer networks show that some minor components, kill larvae, destroy enzyme activity, and disrupt biological glue curing [11]. Modern foul-release coatings take advantage of a loophole in the International Maritime Organization (IMO) convention that permits the use of highly toxic and endocrine-disrupting catalysts, not counting them as biocides. This includes the use of toxic compounds that are not on the lists of known toxins and endocrine disruptors.

To summarize and review knowledge about the interactions between microorganisms and larvae of invertebrates, a Special Issue of the *International Journal of Molecular Sciences* on biofouling and antifouling was published. The main aim of this Issue was to present recent updates in this field and highlight the areas that require more research.

## 2. Review of Publications of the Special Issue

The Special Issue of *IJMS* entitled “Biofouling and Antifouling: Interactions between Microbes and Larvae of Invertebrates” includes five contributions, including two reviews and three research articles. These contributions came from leading scientists in the field working in Japan, Spain, Portugal, the USA, Oman, China, and Ireland. The topics of research publications ranged from the biofouling of steelmaking slags to bacterial flagella genes responsible for larval settlement.

The review by Richards et al. [9] focused on bio-inspired surface texture modifications for antifouling strategies. The review provides a summary of production methods commonly used to produce nano- and micro-scale textured surfaces. The authors indicate that surface wettability, surface roughness, and topography as well as hydrodynamics play an important role in biofouling for a given surface. The review provides a detailed analysis of existing studies on bioinspired biomimetic surfaces that have antifouling potential. However, the authors indicate that most studies demonstrated low antifouling performance and focused on micro-topography of 1–10 μm and 500 μm. Richards et al. [9] indicate that developing complex micro- and nano-scale-level surfaces for antifouling applications is needed.

Dobretsov and Rittschof [5] summarize the current publications about the induction of invertebrate larval settlement and metamorphosis. Their review suggests that the number of publications about larval settlements published in the last 19 years remains low. Most of the studies reviewed were performed on commercially important species, such as barnacles, polychaetes, and corals. Analysis of the published literature suggested that the induction of larval settlement by marine microbes is by the release of inductive molecules, microbial degradation products, and physical viral-like structures found in bacteria. The authors review the induction of larval settlement by monospecies and multispecies bacterial films and the impact of climate change on larval settlement. The authors indicate a limited number of studies on the induction of larval settlement by eukaryotes, which require more attention in the future. A significant part of the review is about bioactive compounds produced by microorganisms, including quorum sensing agents that induce larval settlement. Additionally, molecular aspects of the larval settlement are reviewed. Finally, the authors provide conclusions and highlight future research directions.

The composition of biofilms formed on steelmaking slags was investigated using next-generation sequencing [12]. An artificial sponge was used as a control substrate. The data support the assertion that biofilms are enriched with sulfur-oxidizing bacteria. The observed communities on slags were different from the controls. The authors conclude that steelmaking slags have the potential to be used as artificial seaweed beds and in marine water purification [12].

Cacabelos et al. [13] investigate the role of biofilms in the formation of macrofouling communities. Researchers used biofilms developed in marine protected areas (MPAs) and human-impacted sites. Then, they transplanted these biofilms to different locations and investigated the formation of macrofouling communities. The authors found that the origin of biofilms affects the formation of macrofouling communities [13]. The presence of some invasive species was suppressed by the biofilms from MPAs. These findings could be important in the conservation and restoration of damaged habitats.

In another study, the inducing activity of the bacterium *Pseudoalteromonas marina* for the settlement of *Mytilus coruscus* is investigated in detail [14]. In particular, the authors look for the regulation of biofilm formation and larval settlement by the bacterial flagellar gene. The authors constructed a flagellin synthetic protein gene *fliP* deletion mutant of *P. marina* and investigated its biological properties. From the data, the authors suggest that the mutant led to the loss of flagella, enhanced biofilm formation, and changed the biofilm matrix and reduced the mussel larval settlement [14]. These data enhance our understanding of the molecular mechanisms for larval settlement of commercially important *M. coruscus* and bacterial–larval interactions.

## 3. Future Outlook

The publications in this Issue present novel discoveries in microbial–larval interactions. All the research publications have used modern next-generation sequencing techniques to identify microbes and monitor their changes. This highlights the importance of “omics’ methods in future biofouling studies. The reviews highlight the importance of technical discoveries in the creation of novel antifouling materials and surfaces. More attention should be paid to testing such coatings in the marine environment and in actual industrial applications.

Since the publication of this Special Issue, a few publications about this topic have been published. We highlight two of them. A recent review discussed sustainable antifouling solutions [6]. Rittschof et al. [11] investigated the impact of the chemical composition of the polydimethylsiloxane (PDMS) network commonly used as antifouling coatings on barnacle larvae. While PDMS-based coatings are considered non-toxic, the results show that all the components of the coating altered the barnacle enzymatic activity and adhesion strength. This highlights the importance of understanding the impacts of future more-benign antifouling coatings before the coatings gain market share.
